# Colorectal Cancer Patients’ Reported Frequency, Content, and Satisfaction with Advance Care Planning Discussions

**DOI:** 10.3390/curroncol31030092

**Published:** 2024-02-26

**Authors:** Said Hussein, Chloe Ahryung Lim, Thulasie Manokaran, Shireen Kassam, Madalene Earp, Patricia A. Tang, Safiya Karim, Patricia Biondo, Sharon M. Watanabe, Aynharan Sinnarajah, Amy Tan, Jessica Simon

**Affiliations:** 1 Division of Palliative Medicine, Department of Oncology, Cumming School of Medicine, University of Calgary, Calgary, AB T2N 4Z6, Canada; said.hussein@ucalgary.ca (S.H.); shireen.kassam@ucalgary.ca (S.K.); madalene@gmail.com (M.E.); pbiondo@ucalgary.ca (P.B.); asinnarajah@lh.ca (A.S.); 2 Internal Medicine Residency Program, Cumming School of Medicine, University of Calgary, Calgary, AB T2N 4N1, Canada; chloe.lim@ahs.ca (C.A.L.); thulasie.manokaran@ahs.ca (T.M.); 3 Division of Medical Oncology, Department of Oncology, Cumming School of Medicine, University of Calgary, Calgary, AB T2N 4N2, Canada; patricia.tang@ahs.ca (P.A.T.); safiya.karim@ahs.ca (S.K.); 4 Division of Palliative Care Medicine, Department of Oncology, Faculty of Medicine and Dentistry, University of Alberta, Edmonton, AB T6G 1Z2, Canada; sharon.watanabe2@ahs.ca; 5 Division of Palliative Care, Department of Family Practice, University of British Columbia, Vancouver, BC V6T 1Z4, Canada; 6 Department of Family Medicine, Cumming School of Medicine, University of Calgary, Calgary, AB T2N 4N1, Canada

**Keywords:** advance care planning, goals of care, patient perceptions, patient satisfaction, colorectal cancer

## Abstract

(1) Background: This observational cohort study describes the frequency, content, and satisfaction with advance care planning (ACP) conversations with healthcare providers (HCPs), as reported by patients with advanced colorectal cancer. (2) Methods: The patients were recruited from two tertiary cancer centers in Alberta, Canada. Using the *My Conversations* survey with previously validated questions, the patients were asked about specific ACP elements discussed, with which HCPs these elements were discussed, their satisfaction with these conversations, and whether they had a goals of care designation (GCD) order. We surveyed and analyzed data from the following four time points: enrollment, months 1, 2, and 3. (3) Results: In total, 131 patients were recruited. At enrollment, 24% of patients reported discussing at least one ACP topic. From enrollment to month 3, patients reported a high frequency of discussions (80.2% discussed fears, 71.0% discussed prognosis, 54.2% discussed treatment preferences at least once); however, only 44.3% of patients reported discussing what is important to them in considering health care preferences. Patients reported having ACP conversations most often with their oncologists (84.7%) and cancer clinic nurses (61.8%). Patients reported a high level of satisfaction with their ACP conversations, with over 80% of patients reported feeling heard and understood. From enrollment to month 3, there was an increase in the number of patients with a GCD order from 53% to 74%. (4) Conclusions: Patients reported more frequent conversations compared to the literature and clinical documentation. While the satisfaction with these conversations is high, there is room for quality improvement, particularly in eliciting patients’ personal goals for their treatment.

## 1. Introduction

Advance care planning (ACP) is a dynamic process involving multiple evolving conversations between patients, their family, and their healthcare providers (HCPs) to ensure that patients receive care consistent with their wishes and priorities, especially during serious and chronic illness [1,2]. Systematic reviews have described the complexities of comparing ACP outcomes across studies but have largely concluded that ACP may improve “end-of-life communication, documentation of care preferences, dying in preferred place, and health care savings” [3], and that “outcomes for all ACP interventions were predominantly positive” [4]. Despite the evidence showing improved outcomes with ACP, studies have also demonstrated extensive practice gaps in ACP discussion and documentation [2,5,6,7]. Audits of cancer outpatient documentation about patients’ goals of care have observed low rates of discussion documentation (e.g., 7% prior to quality improvement efforts) [4,8]; however, the frequency of documentation may not accurately reflect conversation frequency. As well, how conversations are experienced by patients, as a marker of the quality of ACP conversations, is an important consideration [9]. The current study sought to describe the frequency and content of, and patient satisfaction with, patient-provider ACP conversations.

## 2. Materials and Methods

### 2.1. Context

This prospective cohort study was conducted as part of a larger program of research, the Palliative Care Early and Systematic (PaCES) project, which was focused on developing, implementing, and evaluating an early palliative care pathway aimed at improving outcomes for patients with advanced cancer [10]. This “Living with colorectal cancer: patient and caregiver experience” study (registered at clinicaltrials.gov, identifier NCT03572101) was conducted in two tertiary cancer centers in Alberta, Canada, namely, the Tom Baker Cancer Centre in Calgary and the Cross Cancer Institute in Edmonton. Since 2014, the province-wide healthcare system has used the Goals of Care Designation (GCD) framework of medical orders to communicate a patient’s general focus of care and the interventions and locations of care that might be used in service of their goals, and to define policy and procedure for ACP and transfer of information between care settings [11]. Provincial cancer practice guidance, introduced in January 2019, encourages clinicians to have ACP conversations, along with a GCD order when appropriate, with patients with advanced colorectal cancer throughout their cancer treatment [12].

### 2.2. Participants

Patients with advanced colorectal cancer, defined as primary or metastatic colorectal cancer that is unlikely to be cured, controlled, or put into remission with treatment, were recruited from medical oncology clinics focused on gastrointestinal cancer. Patients were invited to participate by their care provider if they were >18 years of age and met at least one of the following inclusion criteria: failed first line of chemotherapy (e.g., disease progression on imaging), unable to receive first line chemotherapy, less than one-year prognosis anticipated by the HCP, or reported high symptom burden (Edmonton Symptom Assessment System Revised (ESAS-r) score ≥ 7) [13]. Language spoken was not an exclusion criterion, with translation available for all study materials. Participant recruitment began in January 2018 and continued until June 2020. Data collection was completed in December 2020.

### 2.3. Survey Instruments

The *My Conversations* survey [Supplementary Materials File S1] asks respondents if they had conversations with HCPs in the previous month about the following ACP topics: (1) what was important to them as it relates to health care preferences (values, wishes, goals, spiritual beliefs), (2) prognosis (life expectancy, course of illness), (3) fears and concerns, and (4) treatment preferences. These ACP topics were rated as most important by patients with a life-limiting illness [14] and *My Conversations* uses validated questions from the Audit of Communication, Care Planning, and Documentation (ACCEPT) Study survey [6]. Participants recorded if they were unable to meet with their HCP or if they had no ACP conversations with their HCP. *My Conversations* also asks respondents what type of provider they had ACP conversations with, their level of satisfaction with these conversations (5 = “very satisfied,” 1 = “not at all satisfied”), and if they had a GCD order. Participants also completed a demographic survey at enrollment (age, gender, race, education, marital status, income, religiousness).

### 2.4. Data Collection

Participants were surveyed at enrollment, monthly to month 10, and then every 3 months until death or study end. The time interval between surveys was increased after month 10 to reduce burden to patients. Surveys were completed digitally, or by phone, or in-person with an independent researcher, if the patient preferred. Survey responses were anonymous, each participant had a unique study identifier and participants were informed that none of their data would be shared with their HCPs.

### 2.5. Data Analysis

All data variables were summarized using counts and percentages. We calculated the median (IQR) number of surveys collected per participant and determined the cumulative percentage of participants “off study” by survey month. Because of rising numbers of patients “off study” by month 3, we chose to analyze participants’ cumulative experience with ACP conversations by month 3. Conversation occurrence was defined as discussing an element at least once in a preceding month. We determined the cumulative percentage of participants who discussed each ACP element by survey month (enrollment, months 1, 2, 3). We calculated the percentage of patients reporting each ranking of satisfaction and feeling heard and understood at each survey month. Associations between demographic variables and ACP conversations by month 3 (i.e., ever had versus never had) for each ACP element were tested using chi-square.

## 3. Results

### 3.1. Demographics and Data Collection

A total of 131 participants were enrolled [Supplementary Materials File S2, STROBE diagram]. The median number of My Conversations surveys collected per participant was 8 (IQR 4–11), and 78% of participants were still providing survey data at month 3, the baseline demographics are shown in Table 1. There were more male than female participants; additionally, the majority of participants were married and identified as white, but a diversity of income and educational experience was observed.

### 3.2. Frequency of ACP Discussion and GCD Order Reporting over Time

**Figure 1:**
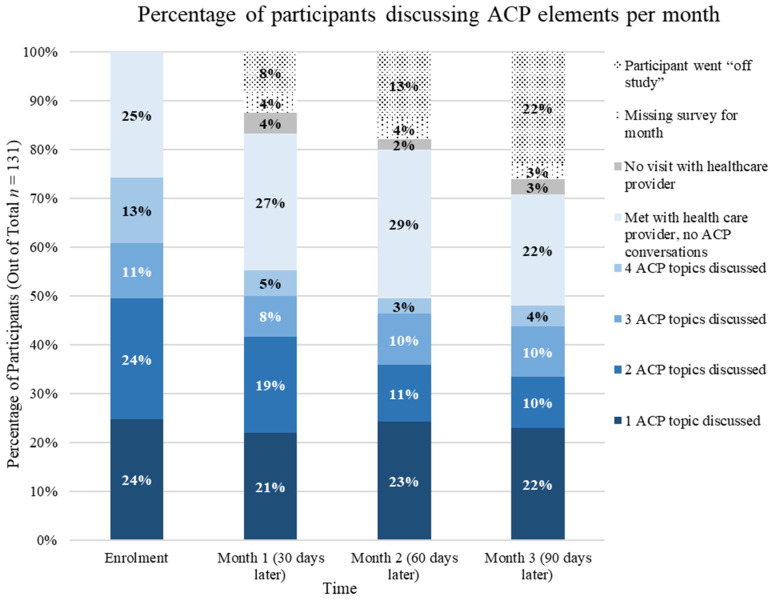
Percentage of participants and number of ACP elements discussed per month (*n =* 131).

At enrollment, 75% of patients reported discussing at least one ACP topic, as seen in Figure 1. One ACP topic was discussed by 24% of patients, 24% discussed two ACP topics, while three and four ACP topics were discussed by 11% and 13% of patients, respectively. One quarter of patients at enrollment reported meeting with their HCP but having no ACP conversations. There was general month-to-month consistency in the percentage of patients discussing one ACP topic, although fewer patients discussed more than one ACP topic over time.

**Figure 2:**
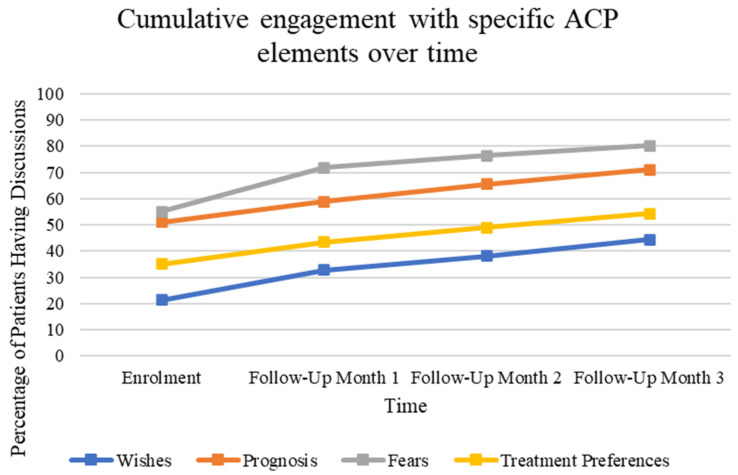
Percentage of patients reporting discussing each of the four surveyed elements of the ACP conversation at the time of enrollment, as well as at three subsequent follow-up appointments. Enrollment *n =* 131, Month-1 *n =* 114, Month-2 *n =* 107, Month-3 *n =* 98.

From enrollment to the third month in follow-up, cumulatively, 91.6% of patients reported discussing at least one ACP element. With regard to specific ACP elements, fears (80.2%), prognosis (71%), and treatment preferences (54.2%) were most often addressed, while important values (44.3%) were the least often discussed by three months (Figure 2).

**Figure 3:**
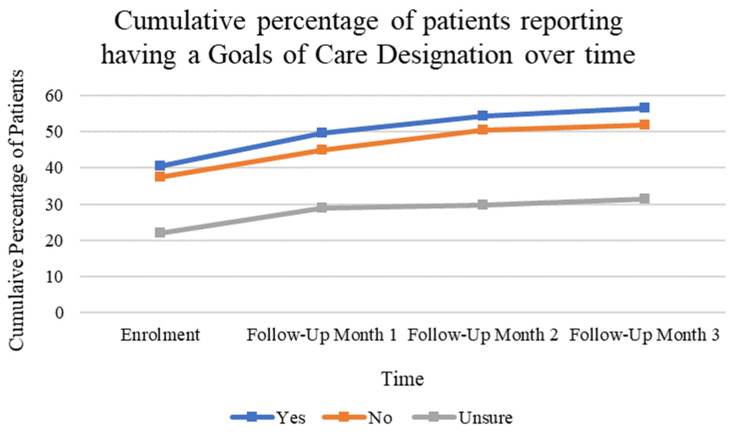
Cumulative percentage of patients reporting having a goals of care designation order at the time of enrollment and at three subsequent follow-up appointments.

From enrollment to the third month of follow-up, patients reported increasing frequency of having a GCD order from 53% to 74%, depicted in Figure 3.

### 3.3. Satisfaction and HCP Participation

**Figure 4:**
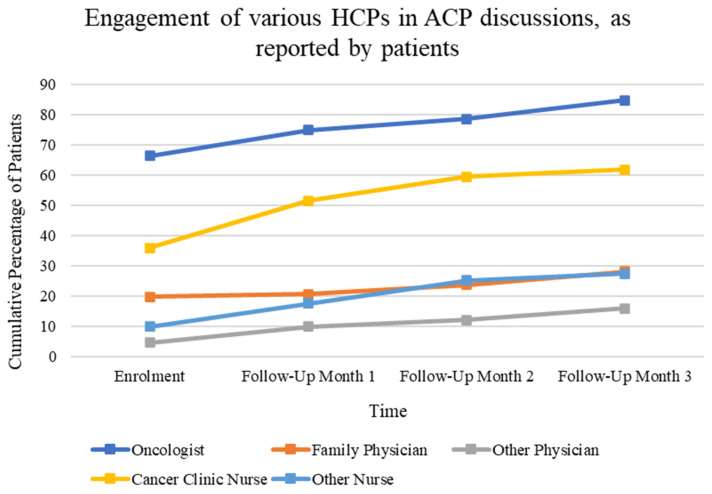
Change over time in the percentage of patients cumulatively reporting having at least one discussion with specified HCPs from enrollment to month 3. Multiple answers were possible at each time frame.

Patients reported having ACP conversations most often with their oncologists (84.7%) and cancer clinic nurses (61.8%). Considerably fewer patients talked to another type of physician (44.2%) or nurse (27.5%) (Figure 4).

**Figure 5:**
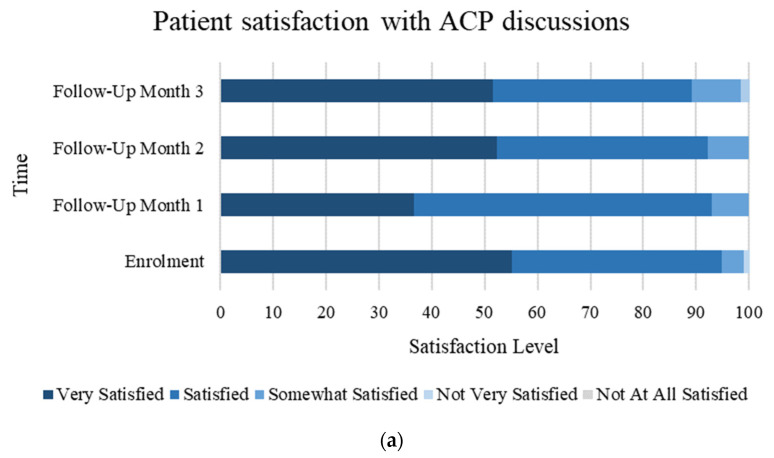

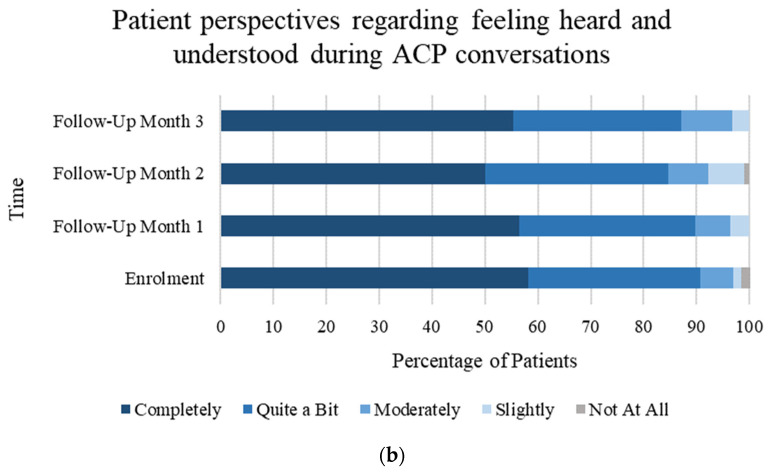
(**a**) Percentage of patients reporting satisfaction with ACP discussions at each listed timepoint. (**b**) Percentage of patients reporting feeling heard and understood during ACP discussions at each listed timepoint.

Furthermore, patients reported a high level of satisfaction with their ACP conversations (over 80% “satisfied” or “very satisfied” each month) (Figure 5a). Patients also reported feeling heard and understood, with over 80% of patients each month feeling “completely” or “quite a bit” heard and understood during ACP conversations (Figure 5b).

During chi-square testing, there were no statistically significant differences in the ACP elements discussed, having a GCD order, and the demographic variables except age. For age, participants ≥70 years old reported lower frequencies of discussing at least one element of ACP discussions than younger adults (76% of ≥70-year-olds discussed at least one element by 3 months, compared to 95% 50–69-year-olds, and 90% 18–49-year-olds, *p* = 0.01). Similarly, discussing treatment preferences differed by age (42% of ≥70 years discussed treatment preferences, compared to 65% 50–69-year-olds, and only 30% of 18–49-year-olds, *p* = 0.005).

## 4. Discussion

This study is notable for describing *longitudinal data* from outpatients living with advanced colorectal cancer reporting their perceived frequency, content, and satisfaction regarding ACP conversations.

The percentage of patients (91.6%) who reported having discussions about at least one ACP element over 3 months is much higher than the literature baseline rates of patient-reported or documented HCP conversations with cancer patients, which ranges from 7.8% to 79% in cross-sectional studies [15,16,17,18,19,20]. Our focus on the colorectal cancer population, which has the second highest mortality rate among all cancers in Canada, may have biased our results toward higher reported rates of ACP discussions than the general cancer population [21]. This study cohort, willing to provide recurrent survey data, may be subject to selection bias and differ from the general colorectal cancer population in their desire and ability to engage their HCPs in ACP conversations. Although direct chart audit was not part of this study, internal health system audits of electronic health record documentation reveal only about 16% of all colorectal outpatients having a GCD order uploaded on the system [22]. Some conversation details may be buried in clinic notes, but baseline data from a quality improvement project in two medical oncology clinics at the Calgary site found rates of documentation on the “ACP GCD tracking record” (the health system designated location for these conversation elements) was as low as 0–4.2% [4]. There thusly appears to be a considerable discrepancy between the rates of ACP discussions as reported by these patients versus what is documented by HCPs in the medical records. Others have reported a difference between what patients perceive they have discussed and what HCPs have documented in health records [23]. The reliability and validity of measurements is one of the challenges of ACP studies [8], but patient-reported outcomes and experiences are important measures in the process of ACP as a complex, relational, and subjective form of communication. Older age was associated with lower conversation frequency; both patient and HCP biases and preferences for communication may explain the variation seen [24].

Regarding specific conversation elements, patients reported that their fears (80.2%) and prognosis (71.0%) were most often discussed, while treatment preferences (54.2%) and important values (44.3%) were less frequently addressed. These are much higher reported frequencies than from older, adult inpatients with a range of serious chronic illnesses (9.8–22.5%) [13], but there is limited comparable data from cancer outpatient studies. Canadian radiation oncologists report routinely discussing prognosis (57%), goals of care (57%), advance directives (40%), and preferred site of death (12%) with patients on palliative intent therapy [25]. Though this result is in keeping with our findings of prognosis being one of the most frequently discussed elements, this has not been consistently demonstrated in other studies. Geerse and colleagues recorded patient–physician encounters and examined oncology clinicians’ adherence to a serious illness conversation guide, which explored eight conversation elements, including prognosis and goals. They found that while goals were discussed in 80% of conversations, only 40% addressed prognosis at all [26]. It is concerning in our study that patients’ important values were least often discussed over three months compared to other ACP elements; this indicates a potential target for quality improvement initiatives. The implementation of the Serious Illness Care Program can increase oncologist documentation of all these key conversation elements, with a randomized controlled trial demonstrating higher rates of the documentation of values and goals (from 44% in control arm to 89% in intervention arm), prognosis and illness understanding (from 48% to 91%), and life-sustaining preferences (from 32% to 63%) [20]. A systematic review of heterogeneous interventions to improve process indicators, however, found limited effects and highlights that interventions that target multiple stakeholders (patients, family caregivers, and HCPs) may be most successful [27].

Participants reported that the majority of ACP conversations at enrollment were held with oncologists (66.4%) and cancer clinic nurses (35.9%), confirming the inter-disciplinary nature of ACP processes [28]. By the final follow-up period, patients reported a much higher frequency of involvement of oncologists (84.7%) and cancer clinic nurses (61.8%) in ACP conversations, as opposed to family physicians (from 19.8% at enrollment to 28.2% at month 3). This could reflect the frequency of cancer center visits compared to family physician contacts over the timeframe studied. This also indicates the need to ensure conversation content is shared across care sectors to provide continuity of patient experience.

The majority of our study’s patients reported they were either ‘very satisfied’ or ‘satisfied’ with ACP conversations throughout the follow-up period and the percentage reporting feeling heard and understood remained fairly constant. We were unable to test for an association between these factors and the number of conversation elements discussed due to small cohort size. Satisfaction with HCP communication has been previously described to correlate with the number of ACP elements discussed [13] and feeling heard and understood is responsive to communication interventions [29].

A strength of our study design was collecting patients’ iterative experiences of ACP conversations. As mentioned above, generalizability from our sample is somewhat limited. Another limitation is that while all patients had advanced colorectal cancer, they were enrolled at different times relative to diagnosis and disease progression. This may have influenced their visit frequency and ACP content discussed over time. From qualitative evidence of conversations with their HCPs, it is known that patients’ needs and desires are dynamic and change over the trajectory of their illness [30,31,32]. HCPs also vary the content of their discussions across the illness trajectory [33]. There are multiple social, emotional, and contextual challenges for ACP in cancer care [34] and even with engagement in ACP, in-the-moment decision-making in cancer emergencies can be fraught and distressing [35]. Another weakness of this study is that we did not ask patients about whether their conversations included family members or their substitute decision maker(s). Making time and space to elicit what is most important to patients, and encouraging the sharing of this with their surrogate decision makers and family are important components of high-quality ACP [36].

Despite these limitations, our results provide fresh insight into patient-perceived frequency of discussion of specific ACP elements. Recognizing that ACP is achieved through a process of iterative conversations and that content changes over time, collecting measures longitudinally may provide a more complete picture of clinical activity than what cross-sectional measures allow. Longitudinal, patient-reported measures may be particularly useful in evaluating interventions to improve communication, such as the Serious Illness Care Program [37] or PREPARE For Your Care [38]. Future studies could explore the longitudinal congruency between patient-perceived rates of ACP discussion and rates of discussion as documented in their medical records, as well as the similarities and differences in what patients and their HCPs find meaningful to discuss and to document during clinical encounters.

## 5. Conclusions

Patients with advanced colorectal cancer were surveyed regarding frequency of discussions of key ACP elements previously reported to be important to patients, including their values, fears, prognosis, and treatment preferences. Over time, the patients in our study reported higher rates of discussion of these elements than what is often reported in the existing literature, with fears and prognosis being more frequently discussed than treatment preferences and important values. Patients reported a high satisfaction and felt heard and understood during their conversations. Measuring the quality and determining the outcomes of ACP interventions remains challenging. Longitudinal patient-reported frequencies of discussions of individual ACP elements, as studied here, may serve as important outcomes to evaluate, during quality improvement or research interventions that aim to enhance engagement or quality of ACP communication over time.

## Figures and Tables

**Table 1:** Demographics of patient cohort.

Demographics	Total (*n* = 131)
**Age at enrollment, median (IQR)**	64 (57–70)
**Gender**	
Female	57 (44%)
Male	74 (56%)
**Race**	
White	115 (88%)
Other	16 (12%)
**Educational attainment**	
<High school	14 (11%)
High school	26 (20%)
College (some or complete)	40 (31%)
University (any level)	51 (39%)
**Marital status**	
Married, or living as married	100 (76%)
Widowed	7 (5%)
Never married	12 (9%)
Divorced, separated, not married	12 (9%)
**Residence prior to admission**	
Own home	128 (98%)
Retirement residence	2 (2%)
Other	1 (1%)
**Income (Canadian dollars)**	
<$25,000	14 (11%)
$25,000–$49,000	19 (15%)
$50,000–$74,999	17 (13%)
$75,000–$99,999	15 (11%)
$100,000	32 (24%)
Decline	34 (26%)
**Patient religiousness**	
Spiritual and religious	45 (34%)
Spiritual but not religious	48 (37%)
Religious but not spiritual	3 (2%)
Neither religious nor spiritual	25 (18%)
Unsure	9 (7%)
Decline	2 (2%)

Some percentages do not add up to 100 due to rounding to the nearest whole number.

## Data Availability

Restrictions apply to the availability of these data. Study participants did not provide consent to share their data, so raw data cannot be shared. Access to anonymized summary level (aggregate data) may be granted by contacting the corresponding author.

## Supplementary Materials

The following supporting information can be downloaded at: https://www.mdpi.com/article/10.3390/curroncol31030092/s1, File S1: My Conversations survey; File S2: STROBE diagram.
