# Supplementary material for: Colorectal Cancer Patients’ Reported Frequency, Content, and Satisfaction with Advance Care Planning Discussions
Source: Curr Oncol. 2024 Feb 26;31(3):1235–45. doi: 10.3390/curroncol31030092 (PMC10969091; doi:10.3390/curroncol31030092)
Supplement: Supplementary file 1 [file curroncol-31-00092-s001.zip › File S1 - My Conversations Survey.pdf]

CUMMING SCHOOL OF MEDICINE

Department of Oncology
Tom Baker Cancer Centre
1331 29th St. NW
Calgary, AB, Canada T2N 4N2
ucalgary.ca

MY CONVERSATIONS

Living with Colorectal Cancer: Patient and Caregiver Experience

**Subject ID**	**Date form completed**
☐☐☐☐☐	___ ___ ___ ___ /___ ___ /___ ___<br>Y Y Y Y M M D D

Some people talk with their healthcare providers (e.g. doctors, nurses, etc.) about their illness, healthcare preferences, and planning for the future.

1. In the last month, has a healthcare provider talked with you about the following? (Check (√) all that apply)

❑ Asked you what is important to you as you consider your healthcare preferences (such as your values, wishes, goals, or spiritual beliefs)

❑ Talked to you about your prognosis (life expectancy, predicted course or outlook of your illness)

❑ Given you the opportunity to express your fears or to discuss what concerns you

❑ Asked you about treatments you prefer to have or not have if you were to be very sick or at the end of life

❑ I had contact with a healthcare provider in the last month but I had none of these conversations **(Go to question 2)**

❑ I did not have contact with a healthcare provider in the last month **(Go to question 3)**

1a) Who did you talk with (Check (√) all that apply)

Doctor

- ❑ Family Doctor
- ❑ Oncologist (Cancer doctor)
- ❑ Other doctor

Nurse

- ❑ Family doctor office nurse
- ❑ Cancer clinic nurse
- ❑ Home visit nurse
- ❑ Other nurse

Other healthcare provider (e.g. social worker, counselor, radiation technician etc.)

❑_please specify ________________________

1b) In general, how satisfied were you with these conversations?

- ❑ Very satisfied
- ❑ Satisfied
- ❑ Somewhat satisfied
- ❑ Not very satisfied
- ❑ Not at all satisfied

Subject ID ☐☐☐☐☐

2. How much do you feel heard and understood by your healthcare providers <u>in the last month</u>?

- ❑ Completely
- ❑ Quite a bit
- ❑ Moderately
- ❑ Slightly
- ❑ Not at all

A Goals of Care Designation is a medical order that guides your healthcare providers about the general focus of your care, the kinds of treatments that might be used, and where you might want that care. You might know it as a "resuscitative", "medical", or "comfort care" order that may be kept in your "green sleeve".

3. Do you have a Goals of Care Designation order?

- ❑ Yes
- ❑ No
- ❑ Unsure

3a) IF YES, to the best of your knowledge, what is the focus of your Goals of Care Designation order?

- ❑ Resuscitative care (R1, R2, R3)
- ❑ Medical care (M1 or M2)
- ❑ Comfort care (C1, C2)
- ❑ Unsure
