# Supplementary material for: Colorectal Cancer Patients’ Reported Frequency, Content, and Satisfaction with Advance Care Planning Discussions
Source: Curr Oncol. 2024 Feb 26;31(3):1235–45. doi: 10.3390/curroncol31030092 (PMC10969091; doi:10.3390/curroncol31030092)
Supplement: Supplementary file 1 [file curroncol-31-00092-s001.zip › File S2- STROBE Diagram.pdf]

**File S2. STROBE flow chart for the Living with Colorectal Cancer Study (My Conversations Survey Completion)**

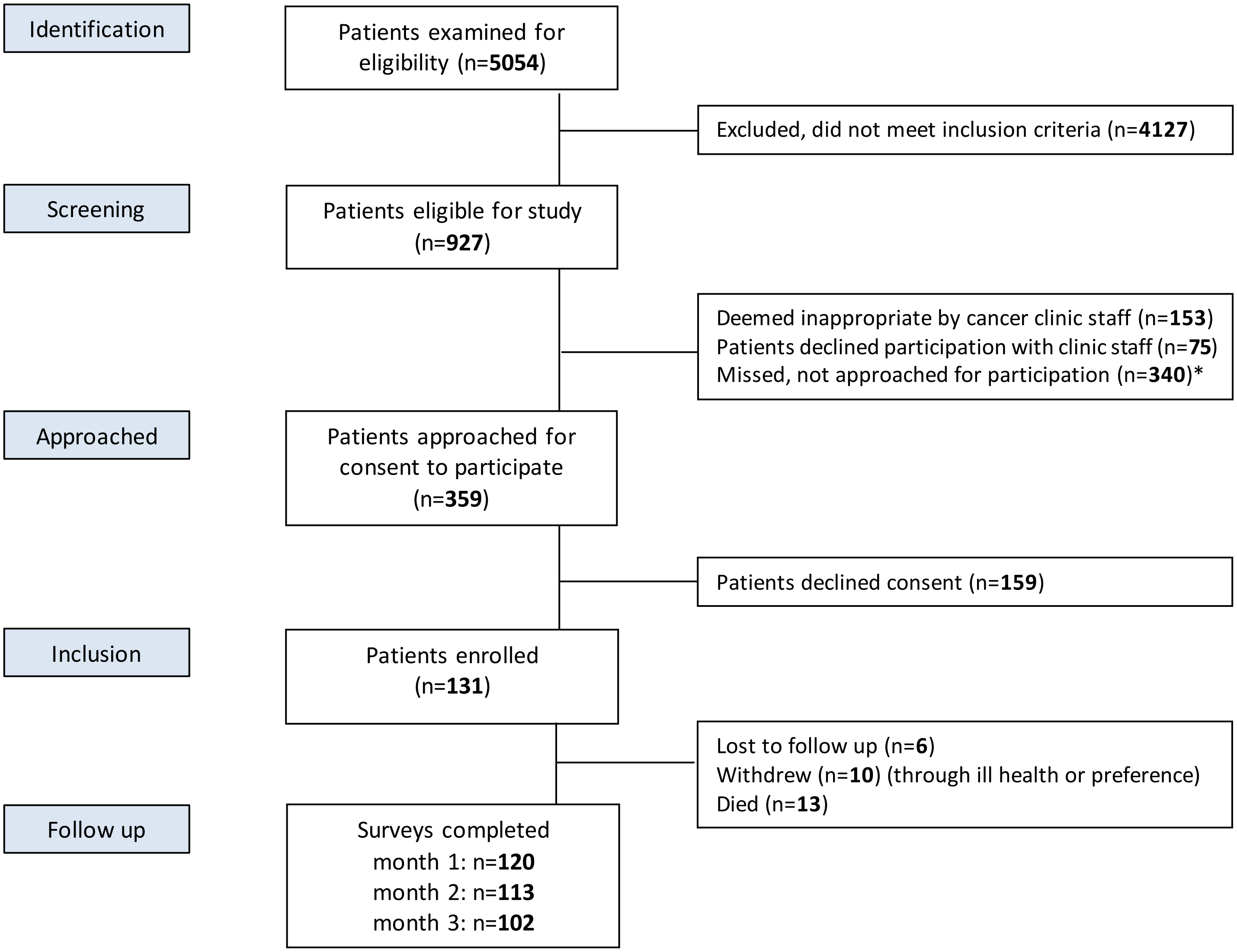


***** Some patients were not approached for participation, particularly when research staff were not in the clinic to prompt and remind clinicians to introduce the study. This was a considerable issue after March 2020 when research staff were unable to be present in the clinic due to COVID-19 pandemic restrictions.
